# Knowledge, Attitudes and Practices (KAP) about Rabies Prevention and Control: A Community Survey in Tanzania

**DOI:** 10.1371/journal.pntd.0003310

**Published:** 2014-12-04

**Authors:** Maganga Sambo, Tiziana Lembo, Sarah Cleaveland, Heather M. Ferguson, Lwitiko Sikana, Cleophas Simon, Honorati Urassa, Katie Hampson

**Affiliations:** 1 Boyd Orr Centre for Population and Ecosystem Health; Institute of Biodiversity, Animal Health and Comparative Medicine; College of Medical, Veterinary and Life Sciences, University of Glasgow, Glasgow, United Kingdom; 2 Ifakara Health Institute, Ifakara, Morogoro, Tanzania; 3 Temeke Municipal Council, Livestock Office, Ministry of Livestock and Fisheries Development, Dar Es Salaam, Tanzania; The Global Alliance for Rabies Control, United States of America

## Abstract

**Background:**

Despite being entirely preventable, canine rabies still kills 55,000 people/year in developing countries. Information about local beliefs and practices can identify knowledge gaps that may affect prevention practices and lead to unnecessary deaths.

**Methodology/Principal Findings:**

We investigated knowledge, attitudes and practices related to rabies and its prevention and control amongst a cross-section of households (n = 5,141) in urban and rural areas of central, southern and northern Tanzania. Over 17% of respondents owned domestic dogs (average of 2.3 dogs/household),>95% had heard about rabies, and>80% knew that rabies is transmitted through dog bites. People who (1) had greater education, (2) originated from areas with a history of rabies interventions, (3) had experienced exposure by a suspect rabid animal, (4) were male and (5) owned dogs were more likely to have greater knowledge about the disease. Around 80% of respondents would seek hospital treatment after a suspect bite, but only 5% were aware of the need for prompt wound cleansing after a bite. Although>65% of respondents knew of dog vaccination as a means to control rabies, only 51% vaccinated their dogs. Determinants of dog vaccination included (1) being a male-headed household, (2) presence of children, (3) low economic status, (4) residing in urban areas, (5) owning livestock, (6) originating from areas with rabies interventions and (7) having purchased a dog. The majority of dog-owning respondents were willing to contribute no more than US$0.31 towards veterinary services.

**Conclusions/Significance:**

We identified important knowledge gaps related to, and factors influencing the prevention and control of rabies in Tanzania. Increasing knowledge regarding wound washing, seeking post-exposure prophylaxis and the need to vaccinate dogs are likely to result in more effective prevention of rabies; however, greater engagement of the veterinary and medical sectors is also needed to ensure the availability of preventative services.

## Author Summary

Rabies remains a major public health problem in Africa and Asia, although means to control and prevent the disease are available through mass dog vaccination and provision of post-exposure prophylaxis to people exposed to bites by suspect rabid animals. Here we report the results of an extensive community survey on knowledge, attitudes and practices related to rabies control and prevention, covering rural and urban settings in central, northern and southern Tanzania. Our results showed that the majority of people across Tanzania had heard about rabies and knew that it is transmitted by dog bites, but most lacked comprehensive knowledge about key practices, such as the need for wound cleansing, which could prevent unnecessary deaths from the disease. In other circumstances, knowledge (for example, about the need to vaccinate dogs to control rabies) did not reflect good practice. In order to address the knowledge gaps identified by this study, there is a need for interventions aimed at increasing awareness, focusing on simple messages and targeting the community as a whole. This information could be channelled through media, community meetings and professionals including community leaders, health workers, teachers, livestock officers and clinicians.

## Introduction

Rabies is one of the oldest recognized infectious diseases, and affects all mammals [1]. The disease is caused by a *rhabdovirus* [2] and is most usually transmitted to humans by domestic dog bites [3]. Canine rabies remains a major socioeconomic and public health problem in developing countries, claiming the lives of an estimated 55,000 people each year [4], [5]. Annual incidence of human rabies deaths typically fall between 1 and 6 cases/100,000 people in canine rabies-endemic areas, with an incidence of 4.9 cases/100,000 reported in Tanzania, 2.5 cases/100,000 in Kenya (Machakos District), 2–3 cases/100,000 in India, 5.8 cases/100,000 in Cambodia, and 1.4 cases/100,000 in Bangladesh [6]–[10]. These reported estimates are from active surveillance studies and not from official records, which typically underestimate the disease burden [4], [6], [11]. The high burden of rabies mortality in most developing countries suggests that, despite the existence of effective human and animal rabies vaccines, rabies prevention and control efforts in these settings are inadequate.

Knowledge, attitudes and practices (KAP) studies have been widely used around the world for different applications in public health based on the principle that increasing knowledge will result in changing attitudes and practices to minimize disease burden [12]. For example, in Thailand a KAP study demonstrated the influence that increasing community knowledge on the control and prevention of dengue had on improving practices for its prevention [13]. Other applications of KAP surveys include identifying knowledge gaps, cultural beliefs and behaviour patterns that may pose barriers to controlling infectious diseases [14]–[17], designing relevant public health awareness campaigns [18], [19], and provision of baseline data for planning, implementation and evaluation of national control programmes. In Swaziland, for instance, KAP surveys were used to investigate local communities' understanding of malaria transmission, recognition of symptoms, perceptions of causes, treatment-seeking patterns, and preventive measures and practices in order to provide baseline data for a national malaria control programme [20]. KAP surveys have also been applied to the study of rabies [17], [21]–[24]. However, before the research described herewith, no such studies had been conducted in Tanzania. The motivation behind this study was the need to provide baseline data that would allow the identification of knowledge gaps that may be affecting rabies control and prevention practices in affected communities in Tanzania.

**Figure 1:**
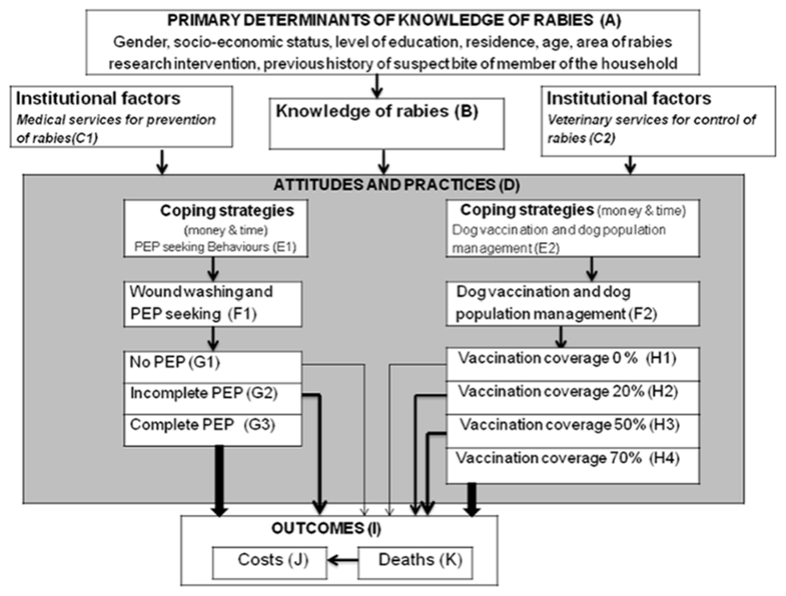
Analytical framework showing how individual knowledge about rabies and institutional factors determine practices for control and prevention. The bolder the arrow the greater the costs incurred but the lower the probability of developing rabies. PEP = Post-exposure prophylaxis.

Human rabies deaths are almost entirely preventable through prompt delivery of post-exposure prophylaxis (PEP) to victims of bites by rabid animals [25] and through successive annual mass dog vaccination campaigns that achieve 70% vaccination coverage to bring rabies under control in reservoir populations [26]–[29]. Thus, for effective rabies control and prevention, veterinary services must coordinate mass dog vaccinations and medical services must provide rabies-exposed individuals with access to PEP (Figure 1, Boxes C1 and C2). However, individuals also need to know the risks associated with rabies and the actions required to prevent human infection, such as seeking PEP when a bite occurs and bringing their dogs to rabies vaccination campaigns. We hypothesized that knowledge about rabies translates into better practices for control and prevention. To address this, we developed a systematic framework, which summarises our hypothesized relationships between rabies knowledge, attitudes and practices, and disease outcomes (Figure 1), for understanding how knowledge may influence rabies control and prevention. Within this framework we investigated existing levels of knowledge, as well as determinants of knowledge and attitudes towards rabies prevention and control in endemic settings of rural and urban Tanzania to determine their influence on practices (Figure 1, Boxes A, B and D). We used data generated from detailed KAP surveys covering over 5,000 respondents in a range of settings across Tanzania, which is the largest KAP survey for rabies that we are aware of in Africa.

## Methods

### Study sites

**Figure 2:**
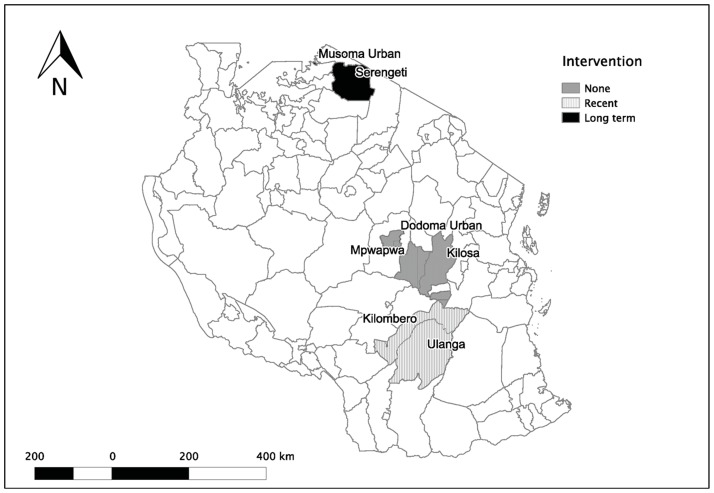
Map of Tanzania showing the study districts. Serengeti and Musoma Urban have had long-term rabies research and control interventions since the 1990s. In Ulanga and Kilombero districts rabies interventions began in 2008, while in Mpwapwa, Kilosa and Dodoma Urban there have been no rabies interventions.

KAP surveys were conducted across Tanzania in seven districts covering approximately 74,748 km^2^, representing 8% of the country's land mass. These areas are inhabited by about 1.8 million people (5.4% of the Tanzanian population according to the 2002 national census [30]). The seven districts were selected to cover three zones (Figure 2) representative of areas with different levels of rabies research and control efforts. The first zone included two districts in northern Tanzania (Musoma Urban and Serengeti) that have been subject to long-term interventions involving mass dog vaccination campaigns and research since the 1990s [6], [26], [27], [31], [32]. The second zone comprised Ulanga and Kilombero districts in southern Tanzania where rabies research and control activities began in 2008. Finally, the third zone included three districts in central-southern Tanzania (Kilosa, Dodoma Urban and Mpwapwa) where no rabies interventions have ever been conducted. The study area represented both urban (Musoma and Dodoma) and rural (other districts) settings. KAP surveys were conducted during two field seasons, August–September 2009 (Ulanga, Kilombero and Kilosa) and April–June 2010 (remaining districts), by nine enumerators who were trained in survey techniques during a pilot study.

In each study district, 25% of villages were selected randomly. Assuming an average household size of 4.9 persons, based on the Tanzania population and housing census [30], we estimated the number of households necessary to survey 5% of the population in the selected villages. Questionnaires were then administered to approximately 5% of households in each surveyed village (a total of 5,141 respondents) after being randomly selected from village households lists.

### Survey methodology

A questionnaire was designed based on consultation with researchers who had conducted KAP surveys elsewhere. The questionnaire was pretested in one village of the survey area and was revised accordingly. The questionnaire was semi-structured with both open and closed-ended questions, and captured details of individual and household characteristics that were used to assess socioeconomic status and education levels. The choice of variables for assessing socioeconomic status followed methodology used by other studies [33], [34]. Additional questions covered (a) knowledge of rabies, including a description of the disease, mode of transmission, outcome, range of species affected and means of prevention and control; and (b) attitudes and practices in relation to rabies prevention strategies and actions towards suspect rabid animals. Further questions were administered to respondents who owned dogs to assess attitudes and practices relevant to rabies control, including willingness to travel to vaccination points, and willingness to pay for dog vaccination.

Research personnel were accompanied by sub-village leaders to identify household heads. Questions were asked to household heads or other household members of at least 18 years of age in the absence of the household head. The questionnaire was conducted in Swahili.

Scores were given according to the completeness and accuracy of respondents' answers, ranging from zero to three depending on the nature of the question [35]–[37] (Appendix S1). For example, regarding the respondent's ability to describe rabies, a score of 2 was assigned if the participant described rabies as a disease, a score of 1 if rabies was described as a change of behaviour and a score of zero if the answer was inaccurate or not provided. If all answers were complete and accurate, a respondent would obtain overall scores of 11 and 10 for (1) rabies knowledge, and (2) attitudes and practices, respectively. For a respondent to be classified as knowledgeable about rabies, a score of 7 or more out of eleven (for knowledge) and 6 or more out of 10 (for attitudes and practices) had to be obtained, which is equal to or more than 60% according to the cut-off point of the Likert-type scale [37]. We also explored other cut-off points (50% to 70%). Binary outcomes were assigned to participants who were knowledgeable and not knowledgeable about rabies, and its prevention and control. Respondents who had dogs were asked if they would be willing to pay for veterinary services such as vaccination and sterilization. If the respondent replied that he/she would, an additional question was asked as to how much he/she would be willing to pay. This question was formulated in the format of a bidding game whereby respondents were asked to bid the maximum amount they would be willing to pay and the answers (maximum/final bid) were recorded [38]. For the purpose of logistic regression analyses, the respondents were classified into two groups: those that were willing or unwilling to pay more than 500 Tanzanian Shillings (TZS) (∼US$ 0.31) to vaccinate each of their dogs. No logistic regression was performed for willingness to sterilize dogs.

### Statistical analysis

Principal component analysis (PCA) was used to estimate the socioeconomic status of respondents (STATA version 10, Stata Corporation; College Station, TX, USA). The possession of household assets was used to calculate mean socio-economic scores for each quintile (least poor, less poor, poor, poorer and poorest [39]. Since the differences in the average scores were small between quintiles we decided to adjoin quintiles into three quintiles (40% corresponding to low economic status, 40% corresponding to medium economic status and 20% corresponding to high economic status) as applied by Filmer and Pritchett [40] and previously used in Tanzania [41], [42].

All other analyses were performed using the R statistical programming language [43]. For categorical explanatory variables (gender, socioeconomic status, education level, residence (i.e. urban/rural dweller) and previous history of bite exposure in the household), frequencies of respondents' answers were compared using Pearson's chi-square test. Pearson's chi-square test was also used to investigate the reported travel time for vaccinations that were charged for versus those that were free-of-charge and in relation to willingness to pay for veterinary services. To determine the influence of all the explanatory variables on each dependent variable (i.e. the level of the respondents' knowledge regarding specific questions), a series of univariate regression analyses were carried out. Variables that were significant in the univariate analysis (p≤0.25) were included in a multivariate analysis. Interaction terms were tested using multiple regression with backward stepwise deletion of non-significant terms (p>0.05). The models were tested for correlations between explanatory variables. Correlations between variables were quantified using *corvif* function of the AED package in R to obtain the variance inflation factors (VIFs) (Table S1) [44]. Logistic regression was used to analyse binary outcome data on whether participants were knowledgeable or lacked knowledge about rabies and whether they reported effective or ineffective practices for rabies prevention and control. Odds ratios (ORs) and 95% confidence intervals (CIs) of binary outcomes about factors that influenced: (a) knowledge about rabies and practices for its prevention and control, (b) dog ownership, (c) vaccination status of dogs and (d) willingness to pay for vaccinating a dog were obtained from the final model. Goodness of fit was examined using the Hosmer-Lemeshow goodness-of-fit test, using the R package *ResourceSelection*.

### Ethics statement

The study protocol was approved by the Medical Research Coordinating Committee of the National Institute for Medical Research of Tanzania (with approval number NIMR/HQ/R.8a/vol.IX/994) and the Institutional Review Board of the Ifakara Health Institute, including the use of oral consent for the collection of data. Written consent was not obtained as the study followed established procedures in Tanzania related to the collection of interview data without collecting biological samples from humans. The study was cleared by the District Executive Director in every study district and the village executive officers were asked for permission prior to the initiation of the research in each village. Before administering questionnaires, participants were orally informed about the purpose of the study, emphasizing that participation was voluntary, and that their answers would be kept confidential. Only participants who verbally agreed were interviewed.

## Results

### Household and individual characteristics including dog ownership

Of the 5,141 respondents (from households covering a population of 27,412), 55% were female with ages ranging from 18–90 (median 35). Most were from rural areas (68%) and the majority (85%) were pastoralists (dependent on grazing livestock), subsistence farmers or engaged in mixed farming practices systems (agro-pastoralists). Most respondents were household heads (61%) and were from areas with no rabies interventions (61%) or areas with recent interventions only (26%). The majority (74%) had only attended primary school, with just 9% having secondary or higher education, whilst 17% had no formal education. Among the respondents without formal education, the majority (73%) were from rural areas (p<0.001) and more were female (69%) than male (p<0.001). Around 8% of households had family members who had previously been bitten by a suspect rabid animal.

Almost one-sixth of households in the survey (17%) owned domestic dogs, with 1–11 dogs per dog-owning household (average 2.3, standard deviation 1.7). The overall human∶dog ratio was 14.3∶1. Dogs were kept for security (78%), for multiple purposes (18%), hunting (4%) and herding livestock (0.5%). About 99% of dog owners fed their dog at least one meal per day, while 78% of households allowed their dogs to roam freely, 6% tied their dogs with a rope during daytime and 15% kept their dogs caged at all times.

### Rabies knowledge

Levels of knowledge about rabies transmission, disease outcome, and prevention in humans and control in animal populations are detailed in the supporting information (Table S2). In brief, the majority (96%) of respondents had heard about rabies. Twenty-seven percent were able to describe rabies as a disease, 41% described it only as a change of behaviour in dogs, and 32% were unable to provide any description. Eighty-one percent knew that rabies was transmitted through bites by suspect rabid animals. While 70% knew that domestic dogs and humans can suffer from rabies, only 7% could name three or more types of animals capable of transmitting rabies. Although the majority of respondents (63%) knew that rabies is fatal following the onset of symptoms, a large percentage was unaware of the fatal nature of the disease. When knowledge of rabies prevention was investigated, 35% of respondents reported that they would expect anti-rabies vaccine at a hospital, 14% reported that they would expect other treatments (e.g. antibiotics, tetanus and pain relief), whereas the rest of the respondents (51%) reported that they would depend on physicians' advice. When asked about methods to control rabies in animals, the majority knew of dog vaccination (mentioned by 67% of respondents), but only 4% knew additional methods such as restraining dogs, and killing suspect animals.

### Determinants of knowledge, attitudes and practices

Of 5,141 respondents, 1,907 (37%) were classified as knowledgeable about rabies. Results of logistic regression (Table 1) indicated that rabies knowledge was greater among respondents (1) with more education, (2) in areas with long-term research interventions, (3) originating from households that had experienced suspect rabid bites, (4) that were male and (5) that owned dogs. There were no significant correlations between any of the variables. Secondary education (and above) was associated with better practices for rabies (Table 1). We explored different cut-offs (50%, 70%) to determine whether respondents where knowledgeable about rabies and its control, but their differences in our findings were negligible.

**Table 1:** Factors influencing levels of knowledge about rabies and rabies control practices in Tanzania (N = 5,141).

Variables		Knowledge¥	Practices‡
		OR (95% CI)	OR (95% CI)
Education background	No formal education	1.00 (reference)	1.00 (reference)
	Primary education	1.90 (1.60–2.25)***	1.29 (1.11–1.50)***
	Secondary education and above	2.33 (1.82–2.97)***	1.58 (1.23–2.04)***
Interventions	No intervention	1.00 (reference)	1.00 (reference)
	Recent intervention	0.95 (1.82–1.10)	1.32 (1.11–1.57)**
	Long term intervention	1.61 (1.35–1.92)***	1.04 (0.87–1.25)
Previous history of exposure	Yes, has exposure	1.56 (1.26–1.93)***	-
	No, has no exposure	1.00 (reference)	-
Socio-economic status	Low status	0.84 (0.72–0.97)**	0.58 (0.50–0.68)***
	Medium status	1.08 (0.92–1.28)	0.68 (0.57–0.81)***
	High status	1.00 (reference)	-
Gender	Male	1.15 (1.02–1.29)*	-
	Female	1.00 (reference)	1.00 (reference)
Dog/Cat ownership	Yes, has either dogs or cats or both	1.30 (1.13–1.49)***	1.76 (1.65–1.88)***
	No, has neither dogs nor cats nor both	1.00 (reference)	1.00 (reference)
Residence	Urban	-	0.84 (0.74–0.96)**
	Rural	-	1.00 (reference)

¥ = Hosmer–Lemeshow goodness-of-fit test, p = 0.4;

‡ = Hosmer–Lemeshow goodness-of-fit test, p = 0.3. Levels of significance:

*** = <0.001,

** <0.01,

* = 0.05.

OR = odds ratio; CI = confidence interval.

### Health seeking behaviours

Following a suspect bite, only 5% of respondents reported that they would apply first aid measures before going to hospital (Table S3). About 95% were not aware of wound cleaning: they claimed that they would report to hospital or to the village leader/police without cleaning the wound or would do nothing. Of the ∼90% of respondents that would seek hospital treatment, 83% claimed that they would seek medical care immediately after a bite, 3% within 2 weeks of being bitten, and 12% after 2 weeks.

### Community actions towards suspect rabid animals

When asked about actions to be taken with regards to a suspect rabid animal, most respondents (79%) reported that they would kill the animal, whereas only 7% would report the incident to a livestock office for further investigation (Table S3). Moreover, only 15 respondents (<1%) were aware that the head of a suspected rabid animal should be submitted to a laboratory for diagnostic confirmation. Most respondents (75%) reported that they would bury or burn the carcass, whereas a minority (25%) stated they would throw away the carcass.

### Factors influencing dog ownership and vaccination

Dog ownership varied significantly across districts in the study and was significantly associated with home ownership, presence of children in households, livestock ownership, and pastoralism, and was negatively associated with farming (Table 2). Determinants of dog vaccination included being a male-headed household, presence of children, low economic status, residing in urban areas, owning livestock, originating from areas with rabies interventions (recent or long-term) and having purchased the dog as opposed to having obtained it for free (Table 2). Of the 1,907 respondents who were classified as knowledgeable of rabies, 494 (26%) were dog owners. Fifty-one percent of dog owners who were classified as knowledgeable of rabies reported to have vaccinated their dogs against rabies (χ^2^ = 3.09, p = 0.07).

**Table 2:** Determinants of dog and cat ownership, dog vaccination and willingness to pay for dog vaccination based on multivariate logistic regression analyses.

Variable		Dog and cat ownership (N = 5,141) OR (95% CI)	Dog vaccination (N = 824 dog owners) OR (95% CI)	Willingness to pay 500 TZS (N = 824 dog owners) OR (95% CI)
District	Kilombero	1.17 (0.9–1.52)	-	0.24 (0.09–0.60)**
	Kilosa	0.23 (0.18–0.28)***	-	0.1 (0.04–0.24)***
	Mpwapwa	0.98 (0.77–1.26)	-	2.14 (1.34–3.45)**
	Musoma Urban	1.37 (1.01–1.85)*	-	0.96 (0.53–1.72)
	Serengeti	1.72 (1.31–2.25)***	-	0.9 (0.56–1.43)
	Ulanga	0.52 (0.38–0.72)***	-	0.44 (0.15–1.28)
	Dodoma Urban	1.00 (reference)	-	1.00 (reference)
Livestock	Own livestock	3.86 (3.01–5.02)***	1.79 (1.09–2.99)*	-
	None	1.00 (reference)	1.00 (reference)	-
Occupation	Civil service	1.19 (0.85–1.67)	NS	-
	Farmer	0.6 (0.46–0.8)***	NS	-
	Pastoralist	1.54 (1.18–2.01)***	NS	-
	Business	1.00 (reference)	-	-
Presence of children	Yes	1.43 (1.2–1.71)***	1.39 (1.01–1.93)*	-
	No	1.00 (reference)	1.00 (reference)	-
Home ownership	Owner	1.35 (1.12–1.65)***	-	-
	Tenant	1.00 (reference)	-	-
Dog ownership	Purchased	-	1.36 (1.06–1.76)*	-
	Obtained free of charge	-	1.00 (reference)	-
Intervention	Long-term	-	1.68 (1.23–2.28)***	-
	Recent	-	2.53 (1.71–3.8)***	-
	None	-	1.00 (reference)	-
Gender	Male	-	1.55 (1.13–2.16)*	-
	Female	-	1.00 (reference)	-
Socio-economic status	Low	-	2.66 (1.78–4.02)***	0.89 (0.38–2.00)
	Medium	-	2.33 (1.54–3.57)***	0.42 (0.17–1.00)*
	High	-	1.00 (reference)	1.00 (reference)
Residence	Urban	-	1.35 (1.04–1.77)*	-
	Rural	-	1.00 (reference)	-

Levels of significance:

*** = <0.001,

** <0.01,

* = 0.05.

NS = not significant. OR = odds ratio; CI = confidence interval. TZS = Tanzanian Shillings.

### Willingness to pay for rabies control

**Figure 3:**
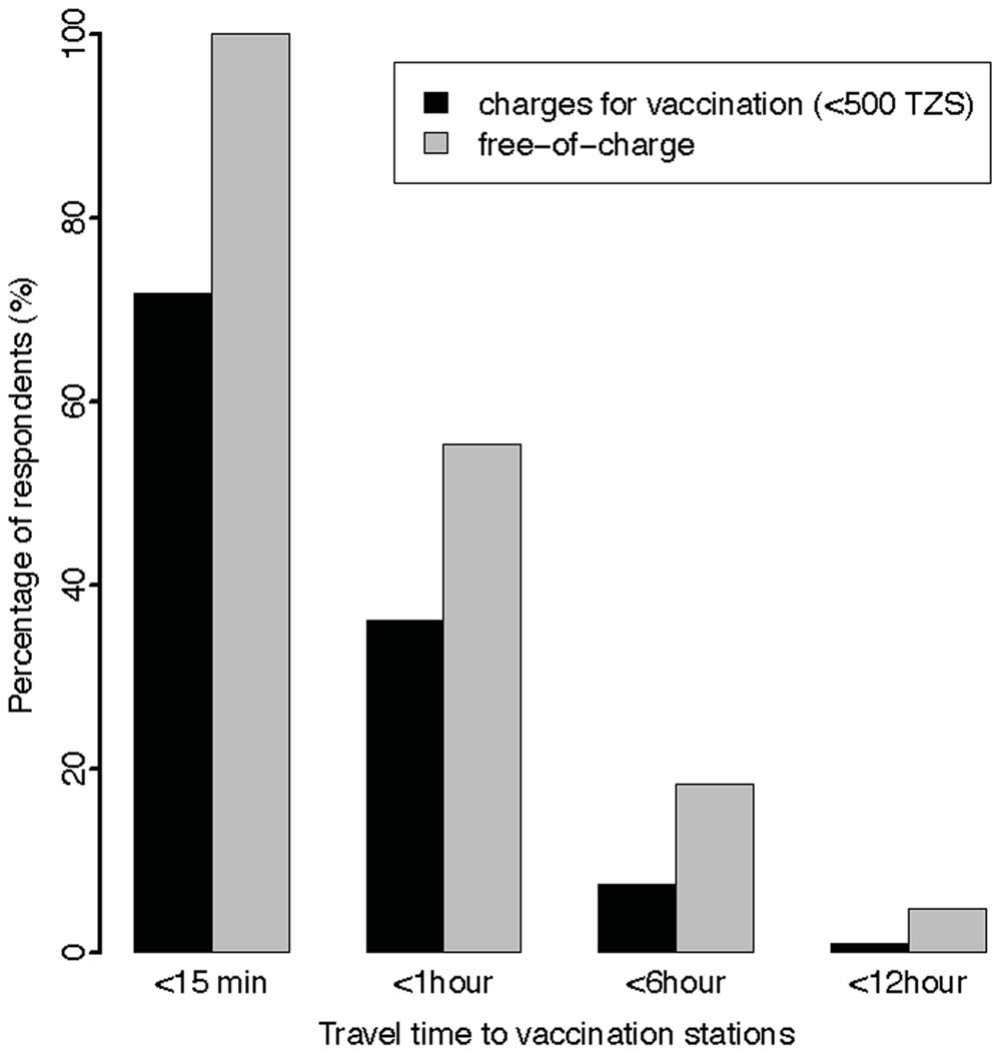
Comparison of reported willingness of respondents' to travel to central point dog rabies vaccination stations when vaccination is offered free of charge or at a cost.

The majority of dog-owning respondents stated their willingness to pay for rabies control including dog sterilization (by surgical or chemical means). However, over half reported that they would not pay more than 500 TZS (Table 3) and very few indicated that they would pay more than ∼US$ 0.31. Instead, if required to incur greater costs, most respondents stated they would opt to vaccinate fewer of their animals. Imposing charges slightly affected reported travel distances to utilise the offered veterinary service with significantly greater willingness to travel further if vaccination was provided for free (Figure 3).

**Table 3:** Reported willingness of dog owners (N = 824) to pay for veterinary services.

Intervention	Vaccination N (%)			P	Surgical operation N (%)			P	Chemical operation N (%)			P
Amount (TZS)	100–500	501–1000	>1000	<0.005	100–500	501–1000	>1000	<0.005	100–500	501–1000	>1000	= 0.05
Long-term, N = 218 (26%)	111 (30)	44 (24)	39 (41)		59 (19)	34 (23)	36 (40)		46 (23)	24 (14.5)	34 (23)	
Recent, N = 123 (15%)	53 (14)	24 (13)	0 (0)		79 (25.5)	17 (11.5)	5 (5)		25 (12)	30 (18)	31 (21)	
None, 483 (59%)	207 (56)	113 (62)	56 (59)		172 (55.5)	97 (65.5)	50 (55)		131 (65)	112 (67.5)	84 (56)	

A total of 647 (76%), 434 (53%), and 544 (66%) respondents reported that they were willing to let their dogs be vaccinated, and either surgically or chemically sterilized, respectively. P = P-values; N = Number. TZS = Tanzanian shillings.

### Sources of information about rabies

The most common source of information about rabies was through personal contacts (neighbours, parents and friends, 70%), while 15% of respondents received information from the media (television, radio and newspapers) and 12% from professionals such as health workers (for bite patients), researchers (during their research activities), or teachers at school. The remaining respondents (3%) knew about rabies from other sources (e.g. leaflets). Multivariate analysis showed that residing in areas with interventions and being of high socioeconomic status influenced the sources of information through which respondents knew about rabies (p<0.001 and p<0.007, respectively).

## Discussion

Rabies remains an important public health problem in Tanzania, where canine rabies is not controlled on a large scale, and the bite of an infected dog is the most common means of transmission. To our knowledge this is the first KAP study on rabies conducted in Tanzania and the largest in sub-Saharan Africa. Our findings highlight key factors affecting rabies knowledge, attitudes and practices across Tanzanian communities that could be targeted to improve health-seeking behaviour and rabies control practices.

The majority of study participants were aware of rabies as a disease and its transmission through bites from infected dogs, and many showed an understanding of the need to seek medical attention following a dog bite and to vaccinate dogs against rabies. But overall there was a lack of comprehensive knowledge about rabies and its prevention, such as the importance of wound washing, the risk of rabies transmission from species other than dogs, and, amongst dog-owners, the need to vaccinate their dogs against rabies. Furthermore, poor awareness about the fatal nature of rabies and how it can be prevented in humans (wound cleaning and seeking medical care for PEP) suggests that human deaths are likely occur due to a lack of knowledge.

Factors influencing better knowledge and practices included higher socioeconomic status and education indicating that the greatest risk of developing rabies is likely to fall on the most vulnerable sectors of society, especially poor members with little or no formal education. Awareness raising interventions aimed at addressing critical knowledge gaps should therefore be targeted to reach all sectors of the population, including those in more marginalized communities. While the value of school-based interventions to increase rabies awareness has been advocated in some settings [45], [46], our study suggests that these interventions are likely to be more effective if combined with community-based programmes involving all members of the community. Our study focused on household decision-makers assumed to be the household head, and we did not survey children, who are disproportionately affected by rabies and often bring dogs for vaccination. Future research among children might inform the design and implementation of interventions targeted specifically at this audience.

Although the majority of respondents had heard about rabies and were aware of its transmission through dog bites, they lacked knowledge regarding certain practices and risk factors. One quarter of respondents claimed that they would throw away the carcass of a rabid animal, a common practice in rural Africa, which has been speculated to be a risk for the spread of rabies to scavengers [47]. Very few respondents knew that rabies could be transmitted by species other than domestic dogs. This is consistent with findings from a survey in Thailand which found that only 16% of participants knew that all mammals can suffer from rabies [48]. Educational information should indicate that all mammals suffer from rabies and can transmit infection and that carcasses should be burned or buried [47].

A critical component of PEP is immediate washing of the bite wound with water and soap before hospital presentation [49]. Our results showed that most respondents were unaware of this preventive practice, which is consistent with other studies [11], [50]–[52]. Lack of wound washing has been shown to be responsible for a five-fold increase in the risk of developing rabies [53]. Improved awareness on wound management (especially prompt flushing with any liquid available) could therefore have considerable impacts on reducing the probability of developing rabies in these communities.

Another essential step following exposure is prompt administration of PEP. In our study 10% of respondents reported that they would not seek medical attention, whereas previous studies in Tanzania have found that around 25% of bite victims do not seek medical attention [53], [54]. This highlights a limitation of our study, which is that our findings correspond to knowledge of correct practices rather than actual behavior. Nonetheless, we show that a substantial proportion of the population is at risk of rabies because they do not have sufficient knowledge of its prevention, which is an area that needs to be addressed to prevent unnecessary deaths. Of the respondents who reported that they would seek treatment, more than half would rely on advice by the health practitioner. Health professionals' knowledge has also been shown to be a major determinant of appropriate treatment for patients [55]. The reported low levels of knowledge about zoonotic diseases among health practitioners in Tanzania, especially in rural areas [56], is cause for concern due to the potential for incorrect advice on post-exposure prophylaxis.

Mass dog vaccination is the most effective measure to control rabies and prevent human deaths [57]. While the majority of respondents knew of the need for dog vaccination, and were willing to vaccinate their dogs, only 46% reported to have previously vaccinated their animals, which reflects a lack of rabies control programmes. Indeed, most respondents reported that dog vaccinations in Tanzania are not regularly conducted except for a few districts where research is undertaken. The majority of respondents also reported that they would be willing to sterilize dogs (both through chemical and surgical means). However, these interventions would be challenging, in terms of both cost and the technical expertise required, and may not be necessary, given that transmission of canine rabies does not appear to depend on dog population density [58], [59].

It is commonly perceived that many African communities are characterised by low levels of responsible dog ownership. Indeed, in this study very few dog owners indicated that they would restrain their animals, indicating that existing legislation is not enforced. However, ‘responsibility’ among dog owners has been clearly demonstrated in this study by the fact that most owners feed their dog, reported a willingness to contribute to dog vaccination costs, and showed an interest in reproductive control of dogs. Responsible dog ownership is therefore likely to reflect the availability and affordability of interventions, as much, if not more than a lack of awareness. Enforcement of rabies legislation or educational programs is likely to be effective only if implemented hand-in-hand with increased availability of affordable interventions, such as vaccination, primary health care or reproductive control.

Although respondents declared a willingness to contribute financially to veterinary interventions, the level of payment is likely to be critical. Willingness to pay studies based on surveys such as this one have limitations as actual behaviour may differ from self-reported claims and is expected to be lower in practice. In West Africa much lower vaccination coverage (∼25%) was achieved when owners were charged in comparison to earlier free vaccinations (68–87%) [60]. The fees charged in West Africa (US$ 1.45) were substantially higher than the levels that owners in Tanzania indicated they could contribute in this study. Given the relatively nominal fees that respondents indicated their willingness to pay for, subsidized mass dog vaccination campaigns should be considered, as the costs of collecting fee could be more expensive than providing free vaccination and would clearly act as a deterrent to many dog owners. This appears to be especially true in areas where free rabies intervention is ongoing (i.e. free mass dog vaccination), but is an important consideration when starting a vaccination programme given that insufficient participation will have limited impact on reducing disease incidence. A lesson learnt from other infectious diseases such as malaria show that policies of subsidised Insecticide-treated bed nets and combination therapy made a great impact on reducing transmission and the number of malaria cases [61]. Indeed Tanzanian dog owners reported that they would be more willing to travel long distances to take their dogs for rabies vaccination if provided for free.

Our study showed that there is generally poor communication between communities and the veterinary sector regarding rabies events in a village. In most cases, respondents reported that they would be prepared to kill suspect rabid animals but would not report to veterinary livestock offices. This makes it difficult for veterinary services to appreciate the scale of the problem and take appropriate steps to prevent further transmission. Awareness messages should focus on informing people that offending animals should be reported to livestock offices. Participatory disease surveillance based on community recognition and reporting of clinical cases provided a wealth of information to direct the last steps of the rinderpest eradication campaigns [62]. Our study and previous studies [31], [58] demonstrate that Tanzanian communities have a sufficient understanding of the presentation of rabies such that community participation in reporting rabies cases would be an important entry point for strengthening rabies surveillance in Tanzania and other rabies-endemic areas.

In recent decades, no national rabies control programs have been implemented in Tanzania, which could probably explain the more limited knowledge about rabies and its prevention and control. It would also explain why most respondents obtained information about rabies from informal channels. The importance of these informal channels again points to the need to consider the development of community-level interventions as a potentially effective approach to increase community and individual awareness, and improve reporting in addition to formal channels such as media, schools and health centres.

### Conclusion and recommendations

Most respondents showed low levels of knowledge about key aspects of rabies and its control and prevention, which should be addressed by key stakeholders. Awareness-raising campaigns focusing on information about the risks associated with rabies and correct behaviour to prevent these risks could prevent unnecessary deaths. Simple messages such as “vaccinate your dogs and cats against rabies”, “immediately wash your wound with water and soap and seek anti-rabies vaccination after a bite from a rabid animal”, “all mammals suffer from rabies”, and “bury or burn carcasses of dead rabid animals”, channelled through government and community networks, could go a long way toward improving community practices. Our recommendations based on experiences in Tanzania could be applied in other developing countries where rabies is endemic.

## Supporting Information

Table S1 **Co-linearity between variables related to rabies knowledge and practices explored using the Variance Inflation Factors (VIFs).**
(DOCX)

Table S2 **Determinants of rabies knowledge (P = P-values obtained using chi-square tests; N = number).** PEP = Post-exposure prophylaxis.
(DOC)

Table S3 **Factors affecting reported practices related to rabies prevention and control (P = P-values obtained using chi-square tests; N = number).**
(DOCX)

Appendix S1 **Questions included in Knowledge, Attitudes and Practices (KAP) surveys in Tanzania to assess (1) knowledge of rabies, its transmission and outcome, species affected, and means of prevention and control; and (2) attitudes and practices towards rabies prevention, and suspect rabid animals and carcasses.** Scores were based on completeness and accuracy of respondents' answers, ranging from zero to three depending on the question.
(DOC)

Checklist S1 **STROBE checklist.**
(DOC)
