# Supplementary material for: Knowledge, Attitudes and Practices (KAP) about Rabies Prevention and Control: A Community Survey in Tanzania
Source: PLoS Negl Trop Dis. 2014 Dec 4;8(12):e3310. doi: 10.1371/journal.pntd.0003310 (PMC4256472; doi:10.1371/journal.pntd.0003310)
Supplement: Table S1 — Co-linearity between variables related to rabies knowledge and practices explored using the Variance Inflation Factors (VIFs). (DOCX) [file pntd.0003310.s001.docx]

**Table S1. Co-linearity between variables related to rabies knowledge and practices explored using the Variance Inflation Factors (VIFs).**

|  | Rabies knowledge | Intervention | Occupation | Sex | Education | Dog ownership | Socio-economic status | Previous history of exposure in the household | Residence |
| --- | --- | --- | --- | --- | --- | --- | --- | --- | --- |
| Rabies knowledge | 1 | 0.01655 | 0.05421 | 0.11829 | 0.01747 | -0.00721 | 0.05965 | 0.02289 | -0.06681 |
| Intervention |  | 1 | 0.02511 | 0.01639 | -0.19450 | -0.26518 | 0.00964 | -0.11527 | -0.00568 |
| Occupation |  |  | 1 | 0.13607 | 0.06587 | -0.01336 | 0.01497 | -0.11003 | -0.07184 |
| Sex |  |  |  | 1 | -0.17081 | 0.07502 | 0.01701 | 0.13338 | -0.03204 |
| Education |  |  |  |  | 1 | -0.16073 | -0.01023 | -0.26768 | -0.04916 |
| Dog ownership |  |  |  |  |  | 1 | -0.00544 | 0.31217 | -0.14464 |
| Socio-economic status |  |  |  |  |  |  | 1 | -0.03413 | -0.02033 |
| Previous history of exposure in the households |  |  |  |  |  |  |  | 1 | -0.05771 |
| Residence |  |  |  |  |  |  |  |  | 1 |
| Age |  |  |  |  |  |  |  |  |  |
|  |  |  | **Variance inflation factors** | | **VIFs** |  |  |  |  |
| Rabies knowledge | 0.009764 |  | Rabies knowledge | | 1.025471 |  |  |  |  |
| Intervention | 0.035184 |  | Intervention | | 1.168335 |  |  |  |  |
| Occupation | 0.145653 |  | Occupation | | 1.080242 |  |  |  |  |
| Sex | -0.19035 |  | Sex | | 1.132677 |  |  |  |  |
| Education | 0.095349 |  | Education | | 1.212277 |  |  |  |  |
| Dog ownership | -0.0223 |  | Dog ownership | | 1.235324 |  |  |  |  |
| Socio-economic status | 0.040513 |  | Socio-economic status | | 1.007862 |  |  |  |  |
| Previous history of exposure in the household | 0.014865 |  | Previous history of exposure in the household | | 1.219207 |  |  |  |  |
| Residence | -0.08389 |  | Residence | | 1.048262 |  |  |  |  |
| Age | 1 |  | Age | | 1.092977 |  |  |  |  |
