# Supplementary material for: Knowledge, Attitudes and Practices (KAP) about Rabies Prevention and Control: A Community Survey in Tanzania
Source: PLoS Negl Trop Dis. 2014 Dec 4;8(12):e3310. doi: 10.1371/journal.pntd.0003310 (PMC4256472; doi:10.1371/journal.pntd.0003310)
Supplement: Table S2 — Determinants of rabies knowledge (P = P-values obtained using chi-square tests; N = number). PEP = Post-exposure prophylaxis. (DOC) [file pntd.0003310.s002.doc]

**Table S2. Determinants of rabies knowledge (P = P-values obtained using chi-square tests; N = number). PEP = Post-exposure prophylaxis.**

| **Knowledge variables**  **(%)** | **Level of education**  **N (%)** | | | **P** | **Rabies intervention**  **N (%)** | | | **P** | **Gender**  **N (%)** | | **P** | **Socioeconomic status**  **N (%)** | | | **P** | **Residence**  **N (%)** | | **P** | **Previous exposure**  **N (%)** | | **P** |
| --- | --- | --- | --- | --- | --- | --- | --- | --- | --- | --- | --- | --- | --- | --- | --- | --- | --- | --- | --- | --- | --- |
|  | **None 858 (17)** | **Primary**  **3817 (74)** | **Secondary & above 466 (9)** |  | **None 3119 (61)** | **Recent 1313 (26)** | **Long-term 709 (14)** |  | **F**  **2811 (55)** | **M**  **2330 (45)** |  | **Low 2056 (40)** | **Medium 1031 (20)** | **High 2054 (40)** |  | **Rural**  **3476 (68)** | **Urban**  **1665 (32)** |  | **No**  **4753 (92)** | **Yes**  **388 (8)** |  |
| ***Description of rabies:*** |  |  |  | <0.001 |  |  |  | <0.001 |  |  | <0.001 |  |  |  | <0.001 |  |  | 0.6 |  |  | <0.001 |
| Correctly described as a disease (27) | 159 (19) | 1084 (28) | 163 (35) |  | 686 (22) | 458 (35) | 262 (37) |  | 679 (24) | 727 (31) |  | 470 (23) | 290 (28) | 646 (31) |  | 944 (27) | 462 (28) |  | 1275 (27) | 131 (34) |  |
| Partially described (41) | 330 (38) | 1543 (41) | 198 (42) |  | 1354 (43) | 417 (32) | 300 (42) |  | 1097 (39) | 974 (42) |  | 923 (45) | 434 (42) | 714 (35) |  | 1391 (40) | 680 (41) |  | 1884 (40) | 187 (48) |  |
| Unable to describe (32) | 369 (43) | 1190 (31) | 105 (23) |  | 1079 (35) | 438 (33) | 147 (21) |  | 1035 (37) | 629 (27) |  | 663 (32) | 307 (30) | 694 (34) |  | 1141 (33) | 523 (31) |  | 1594 (33) | 70 (18) |  |
| ***Mode of transmission:*** |  |  |  | <0.001 |  |  |  | <0.001 |  |  | <0.001 |  |  |  | <0.001 |  |  | 0.03 |  |  | <0.001 |
| Through bites (80.7) | 628 (73) | 3131 (82) | 390 (84) |  | 2432 (78) | 1108 (84) | 609 (86) |  | 2210 (79) | 1939 (83) |  | 1569(76) | 862 (84) | 1718(84) |  | 2839 (82) | 1310 (79) |  | 3783 (80) | 366 (94) |  |
| Through scratches (0.3) | 2 (0) | 12 (0) | 1 (0) |  | 10 (0) | 2 (0) | 3 (0) |  | 5 (0) | 10 (1) |  | 8 (1) | 1 (0) | 6 (0) |  | 11 (0) | 4 (0) |  | 14 (0) | 1 (0) |  |
| Unknown (19) | 228 (27) | 674 (18) | 75 (16) |  | 677 (22) | 203 (16) | 97 (14) |  | 596 (21) | 381 (16) |  | 479 (23) | 168 (16) | 330 (16) |  | 626 (18) | 351 (21) |  | 956 (20) | 21 (21) |  |
| ***Species affected:*** |  |  |  | <0.01 |  |  |  | <0.001 |  |  | 0.03 |  |  |  | <0.001 |  |  | <0.001 |  |  | <0.001 |
| 3 or more species known (4) | 44 (5) | 267 (7) | 34 (7) |  | 197 (6) | 117 (9) | 31 (5) |  | 171 (6) | 174 (8) |  | 111 (5) | 80 (8) | 154 (8) |  | 256 (7) | 89 (5) |  | 308 (7) | 37 (9) |  |
| 1 or 2 species known (83) | 591 (69) | 2695 (71) | 299 (64) |  | 2109 (68) | 1070 (81) | 406 (57) |  | 1947 (69) | 1638 (70) |  | 1183 (58) | 672 (65) | 1730 (84) |  | 2559 (74) | 1026 (62) |  | 3295 (69) | 290 (75) |  |
| Unknown (13) | 223 (26) | 855 (22) | 133 (29) |  | 813 (26) | 126 (10) | 272 (38) |  | 693 (25) | 518 (22) |  | 762 (37) | 279 (27) | 170 (8) |  | 661 (19) | 550 (33) |  | 1150 (24) | 61 (16) |  |
| ***Rabies prevention:*** |  |  |  | <0.001 |  |  |  | <0.001 |  |  | 0.24 |  |  |  | <0.001 |  |  | <0.001 |  |  | 0.04 |
| Expect PEP (35) | 235 (27) | 1368 (36) | 195 (42) |  | 1139 (36) | 328 (25) | 331 (47) |  | 957 (34) | 841 (36) |  | 778 (38) | 411 (40) | 609 (30) |  | 1156 (33) | 642 (38) |  | 1639 (34) | 159 (41) |  |
| Expect medical attention but unaware of PEP (51) | 107 (13) | 541 (14) | 54 (12) |  | 487 (16) | 132 (10) | 83 (12) |  | 397 (14) | 132 (10) |  | 247 (12) | 136 (13) | 319 (15) |  | 526 (15) | 176 (11) |  | 654 (14) | 48 (12) |  |
| Unknown (13) | 516 (60) | 1908 (50) | 217 (46) |  | 1493 (48) | 853 (65) | 295 (42) |  | 1457 (52) | 1184 (51) |  | 1031 (50) | 484 (47) | 1126 (55) |  | 1794 (52) | 847 (51) |  | 2460 (52) | 181 (47) |  |
| ***Rabies control:*** |  |  |  | <0.001 |  |  |  | <0.001 |  |  | <0.001 |  |  |  | <0.001 |  |  | <0.001 |  |  | <0.001 |
| 3 or more methods known (4) | 15 (2) | 140 (4) | 26 (6) |  | 107 (3) | 62 (5) | 12 (2) |  | 88 (3) | 93 (4) |  | 63 (3) | 44 (4) | 74 (4) |  | 106 (3) | 75 (5) |  | 156 (3) | 25 (6) |  |
| 1 to 2 methods known (67) | 449 (52) | 2606 (68) | 406 (87) |  | 2089 (67) | 677 (51) | 695 (98) |  | 1790 (64) | 1671 (72) |  | 1712 (83) | 762 (74) | 987 (48) |  | 2087 (60) | 1374 (82) |  | 3190 (67) | 271 (70) |  |
| Unknown (29) | 394 (46) | 1071 (28) | 34 (7) |  | 923 (30) | 574 (44) | 2 (0) |  | 933 (33) | 566 (24) |  | 281 (14) | 225 (22) | 993 (48) |  | 1283 (37) | 216 (13) |  | 1407 (30) | 92 (24) |  |
| ***Knowledge that rabies is fatal:*** |  |  |  | 0.04 |  |  |  | 0.03 |  |  | 0.94 |  |  |  | =0. 55 |  |  | <0.001 |  |  | 0.69 |
| Known (63) | 569 (66) | 2356 (62) | 297 (64) |  | 1914 (61) | 837 (64) | 471 (66) |  | 1760 (63) | 1462 (63) |  | 1270 (62) | 653 (63) | 1299 (63) |  | 2110 (61) | 1112 (67) |  | 2983 (63) | 239 (62) |  |
