# Supplementary material for: Knowledge, Attitudes and Practices (KAP) about Rabies Prevention and Control: A Community Survey in Tanzania
Source: PLoS Negl Trop Dis. 2014 Dec 4;8(12):e3310. doi: 10.1371/journal.pntd.0003310 (PMC4256472; doi:10.1371/journal.pntd.0003310)
Supplement: Table S3 — Factors affecting reported practices related to rabies prevention and control (P = P-values obtained using chi-square tests; N = number). (DOCX) [file pntd.0003310.s003.docx]

**Table S3. Factors affecting reported practices related to rabies prevention and control (P = P-values obtained using chi-square tests; N=number).**

| **Knowledge variables (%)** | **Level of education N (%)** | | | **P** | **Rabies intervention N (%)** | | | **P** | **Gender N (%)** | | **P** | **Socioeconomic status N (%)** | | | **P** | **Residence N (%)** | | **P** | **Previous exposure N (%)** | | **P** |
| --- | --- | --- | --- | --- | --- | --- | --- | --- | --- | --- | --- | --- | --- | --- | --- | --- | --- | --- | --- | --- | --- |
|  | **None 858 (17)** | **Primary 3817 (74)** | **Secondary & above 466 (9)** |  | **None 3119 (61)** | **Recent 1313 (26)** | **Long-term 709 (14)** |  | **F**  **2811 (55)** | **M 2330 (45)** |  | **Low 2056 (40)** | **Medium 1031 (20)** | **High 2054 (40)** |  | **Rural**  **3476 (68)** | **Urban**  **1665 (32)** |  | **No 4752 (92)** | **Yes**  **388 (8)** |  |
| ***First aid and medical attention:*** |  |  |  | <0.001 |  |  |  | <0.001 |  |  | <0.001 |  |  |  | <0.001 |  |  | <0.001 |  |  | <0.001 |
| Do nothing (10) | 118 (14) | 372 (10) | 33 (7) |  | 338 (11) | 124 (9) | 61 (9) |  | 325 (11) | 198 (8) |  | 283 (14) | 91 (9) | 149 (7) |  | 331 (9) | 192 (12) |  | 509 (11) | 14 (3) |  |
| Report to police/ village leader then go to hospital (4) | 34 (4) | 151 (4) | 22 (5) |  | 162 (5) | 29 (2) | 16 (2) |  | 103 (4) | 105 (5) |  | 114 (6) | 50 (5) | 43 (2) |  | 90 (3) | 117 (7) |  | 189 (4) | 18 (5) |  |
| Report to hospital (81) | 684 (79) | 3094 (81) | 371 (80) |  | 2534 (81) | 1007 (77) | 608 (86) |  | 2282 (81) | 1866 (80) |  | 1607 (78) | 849 (82) | 1693 (83) |  | 2861 (82) | 1288 (77) |  | 3835 (81) | 314 (81) |  |
| Wash wound then go to hospital (5) | 22 (3) | 200 (5) | 40 (8) |  | 85 (3) | 153 (12) | 24 (3) |  | 101 (4) | 161 (7) |  | 52 (2) | 41 (4) | 169 (8) |  | 194 (6) | 68 (4) |  | 220 (4) | 42 (11) |  |
| ***Presentation to hospital after exposure:*** |  |  |  | <0.001 |  |  |  | <0.01 |  |  | <0.01 |  |  |  | <0.001 |  |  | <0.001 |  |  | <0.001 |
| >15 days (12) | 145 (17) | 458 (12) | 45 (10) |  | 433 (14) | 156 (12) | 59 (8) |  | 387 (14) | 261 (11) |  | 339 (16) | 119 (11) | 190 (9) |  | 387 (11) | 261 (16) |  | 625 (13) | 23 (6) |  |
| 2-14 days (3) | 26 (3) | 110 (3) | 6 (1) |  | 85 (3) | 39 (3) | 18 (3) |  | 74 (3) | 68 (3) |  | 56 (3) | 32 (3) | 54 (3) |  | 111 (3) | 31 (2) |  | 117 (2) | 25 (6) |  |
| 1 day (2) | 12 (1) | 82 (2) | 9 (2) |  | 61 (2) | 27 (2) | 15 (2) |  | 43 (1) | 60 (3) |  | 36 (2) | 15 (2) | 52 (2) |  | 80 (2) | 23 (1) |  | 89 (2) | 14 (4) |  |
| On day of bite (83) | 675 (77) | 3167 (83) | 406 (87) |  | 2540 (81) | 1091 (83) | 617 (87) |  | 2307 (82) | 1941 (83) |  | 1625 (79) | 865 (84) | 1758 (86) |  | 2898 (84) | 1350 (81) |  | 3922 (83) | 326 (84) |  |
| ***Action towards suspect biting animal:*** |  |  |  | <0.001 |  |  |  | <0.001 |  |  | <0.001 |  |  |  | <0.001 |  |  | <0.001 |  |  | 0.05 |
| Do nothing (14) | 163 (19) | 509 (13) | 60 (13) |  | 410 (13) | 185 (14) | 137 (19) |  | 473 (17) | 259 (11) |  | 338 (16) | 147 (14) | 247 (12) |  | 460 (13) | 272 (16) |  | 693 (14) | 39 (10) |  |
| Kill the animal (79) | 629 (73) | 3068 (81) | 363 (78) |  | 2484 (80) | 1054 (80) | 522 (74) |  | 2148 (76) | 1912 (82) |  | 1534 (75) | 792 (77) | 1734 (84) |  | 2623 (81) | 1237 (74) |  | 3740 (79) | 320 (82) |  |
| Report to livestock office (7) | 66 (8) | 240 (6) | 43 (9) |  | 225 (7) | 74 (6) | 50 (7) |  | 190 (7) | 159 (7) |  | 184 (9) | 92 (9) | 73 (4) |  | 193 (6) | 156 (10) |  | 320 (7) | 29 (8) |  |
| ***Action towards carcass of biting animal:*** |  |  |  | 0.002 |  |  |  | <0.001 |  |  | <0.001 |  |  |  | <0.001 |  |  | 0.26 |  |  | <0.001 |
| Throw away/ do nothing (25) | 260 (30) | 913 (24) | 109 (23) |  | 783 (25) | 308 (23) | 191 (27) |  | 774 (28) | 508 (22) |  | 604 (29) | 244 (24) | 434 (21) |  | 862 (25) | 420 (25) |  | 1197 (25) | 85 (22) |  |
| Bury or burn (75) | 597 (70) | 2891 (76) | 356 (77) |  | 2333 (75) | 994 (76) | 517 (73) |  | 2032 (72) | 1812 (78) |  | 1450 (71) | 784 (76) | 1610 (78) |  | 2602 (75) | 1242 (75) |  | 3543 (75) | 301 (78) |  |
| Cut head & send to livestock office (0) | 1 (0) | 13 (0) | 1 (0) |  | 3 (0) | 11 (1) | 1 (0) |  | 5 (0) | 10 (0) |  | 2 (0) | 3 (0) | 10 (0) |  | 12 (0) | 3 (0) |  | 13 (0) | 2 (0) |  |
