# Supplementary material for: Knowledge, Attitudes and Practices (KAP) about Rabies Prevention and Control: A Community Survey in Tanzania
Source: PLoS Negl Trop Dis. 2014 Dec 4;8(12):e3310. doi: 10.1371/journal.pntd.0003310 (PMC4256472; doi:10.1371/journal.pntd.0003310)
Supplement: Appendix S1 — Questions included in Knowledge, Attitudes and Practices (KAP) surveys in Tanzania to assess (1) knowledge of rabies, its transmission and outcome, species affected, and means of prevention and control; and (2) attitudes and practices towards rabies prevention, and suspect rabid animals and carcasses. Scores were based on completeness and accuracy of respondents' answers, ranging from zero to three depending on the question. (DOC) [file pntd.0003310.s004.doc]

**Appendix S1 – Questions included in Knowledge, Attitudes and Practices (KAP) surveys in Tanzania to assess (1) knowledge of rabies, its transmission and outcome, species affected, and means of prevention and control; and (2) attitudes and practices towards rabies prevention, and suspect rabid animals and carcasses. Scores were based on** completeness and accuracy of respondents’ answers, ranging from zero to three depending on the question.

| **Question asked** | **Answer** | **Scores** | **Binary outcome** |
| --- | --- | --- | --- |
| **Description of rabies** | Rabies described as a disease | 2 | NA |
|  | Rabies described as change of behaviour of a dog/animal | 1 |  |
|  | Unknown/wrong answer | 0 | NA |
| **Mode of transmission/how rabies can be caught** | Through bites | 2 | NA |
|  | Through scratches | 1 | NA |
|  | Unknown/wrong answer | 0 | NA |
| **Animals that can be infected by rabies** | Three or more animals mentioned | 2 | NA |
|  | One or two animals mentioned | 1 | NA |
|  | Unknown/wrong answer | 0 | NA |
| **Knowledge of treatment when exposed** | Human post-exposure prophylaxis (PEP) | 2 | NA |
|  | Antibiotic and anti-tetanus treatment without mentioning PEP | 1 | NA |
|  | I do not know/advice from medical practitioner sought | 0 | NA |
| **Knowledge that rabies is fatal** | Fatal nature of the disease known | 1 | NA |
|  | Fatal nature of the disease unknown | 0 | NA |
| **Knowledge of control of rabies in animal** | Three or four methods known | 2 | NA |
|  | One or two methods known | 1 | NA |
|  | Unknown/wrong answer | 0 | NA |
| **Knowledgeable of rabies** | Respondents whose score was ≥ 7/11 | ≥ 7 | 1 |
| **Unknowledgeable of rabies** | Respondents whose score was ≤ 6/11 | ≤ 6 | 0 |
| **Overall score** |  | 11 |  |
| **First aid and medical attention** | Respondents that indicated wound cleaning with water, soap, and/or kerosene, as well as subsequently reporting to the hospital | 3 | 1 |
|  | Respondents who would report to the hospital | 2 | 1 |
|  | Respondents who would report to police or village leader | 1 | 0 |
|  | Respondents who would do nothing | 0 | 0 |
| **Hospital presentation after bite** | Respondents who would report to the hospital immediately after a bite | 3 | 1 |
|  | Respondents who would report to the hospital the following day after a bite | 2 | 1 |
|  | Respondents who would report to the hospital 2 to 14 days after a bite | 1 | 1 |
|  | Respondents who would report to the hospital 14 days after a bite or would do nothing | 0 | 0 |
| **Practice towards a suspect rabid animal** | Respondents who would report to the livestock office and kill the animal | 2 | 1 |
|  | Respondents who would kill the animal | 1 | 1 |
|  | Respondents who would do nothing | 0 | 0 |
| **Practice towards the carcass of a suspect animal** | Respondents who would cut the head and send it to the livestock office | 2 | 1 |
|  | Respondents who would bury/burn the carcass | 1 | 1 |
|  | Respondents who would do nothing | 0 | 0 |
